# Inhibition of *Pseudomonas aeruginosa* Biofilm Formation with Surface Modified Polymeric Nanoparticles

**DOI:** 10.3390/pathogens8020055

**Published:** 2019-04-24

**Authors:** Tyler R. Flockton, Logan Schnorbus, Agustin Araujo, Jill Adams, Maryjane Hammel, Lark J. Perez

**Affiliations:** Department of Chemistry and Biochemistry, Rowan University, 201 Mullica Hill Road, Glassboro, NJ 08028, USA; flocktont0@rowan.edu (T.R.F.); schnorbul6@students.rowan.edu (L.S.); Agustin-A-13@hotmail.com (A.A.); jillatoms@outlook.com (J.A.); maryjanehammel@gmail.com (M.H.)

**Keywords:** biofilm, inhibition, antivirulence, lectin, *Pseudomonas aeruginosa*, nanoparticle

## Abstract

The gram-negative bacterial pathogen *Pseudomonas aeruginosa* represents a prominent clinical concern. Due to the observed high levels of antibiotic resistance, copious biofilm formation, and wide array of virulence factors produced by these bacteria, new treatment technologies are required. Here, we present the development of a series of *P. aeruginosa* LecA-targeted polymeric nanoparticles and demonstrate the anti-adhesion and biofilm inhibitory properties of these constructs.

## 1. Introduction

The ongoing increase of antibiotic resistance represents a significant challenge to modern medicine [1,2,3]. Due to the widespread use of antibiotics in clinical and agricultural settings and the limited number of novel antibiotics being advanced into the market, the development of new treatment options for bacterial infection is of paramount importance. 

*Pseudomonas aeruginosa* represents one of the most significant gram-negative opportunistic pathogens involved in hospital-acquired infections. A report from the CDC-NNIS (National Nosocomial Infections Surveillance, US Dept. of Health and Human Services) identifies *P. aeruginosa* as the second leading cause of pneumonia, the third leading cause of urinary tract infection, and the eighth most frequently isolated pathogen from the bloodstream [1,2,3,4,5,6,7]. Further, *P. aeruginosa* often causes lethal infections in cystic fibrosis and immunocompromised patients, for whom the formation of bacterial biofilms plays a central role in infection and resistance [8,9]. Given the rapidly increasing incidence of *P. aeruginosa* in the clinic coupled with the high levels of antibiotic resistance and production of copious biofilms often found with this pathogen, the development of novel treatment strategies is imperative [10,11,12]. 

In *P. aeruginosa*, biofilm formation is regulated by a cell-cell communication mechanism known as quorum sensing, which is mediated by bacterial cell-surface lectins involved in cellular adhesion [13,14]. Specifically, the galactose-binding lectin LecA (PA-IL) and the fucose-binding lectin LecB (PA-IIL) have prominent roles in bacterial virulence, cellular adhesion, tissue colonization, and/or invasion and biofilm formation [15,16,17]. Several recent reports have evaluated approaches to inhibit the virulence and/or biofilm formation in *P. aeruginosa* through inhibition of quorum sensing or lectin binding. Notable about these anti-virulence approaches is the potential they possess for species-selectivity by targeting the inhibition of cellular processes in a manner that uniquely inhibits the virulence and/or infectivity of *P. aeruginosa*. Accordingly, treatments developed for these *P. aeruginosa*-specific targets are anticipated to have a lower impact on the beneficial commensal bacterial population and may possibly exhibit reduced pressure for the development of antibiotic resistance.

Prior studies developing inhibitors of *P. aeruginosa* LecA have described mono-valent and multi-valent inhibitors of this lectin and have characterized the positive effect on inhibitor avidity through multivalency [12,17]. We rationalized that we might achieve similar high binding avidity for LecA using a polymeric nanoparticle with multiple copies of a LecA ligand on the surface of the particle. Further, we recognized that unlike dendrimeric or small molecule inhibitors of LecA, the surface-modified polymeric nanoparticles that we aimed to prepare would be capable of encapsulating a fluorescent or drug molecule within their lipophilic core, potentially enabling future applications in targeted antibiotic drug delivery or fluorescent labeling for diagnostic applications. Here we report our findings on the development and anti-biofilm properties of a series of LecA-targeted polymeric nanoparticles. 

## 2. Results

### 2.1. Synthesis of Galactose-Modified Di-block Co-polymer

**Scheme 1:**
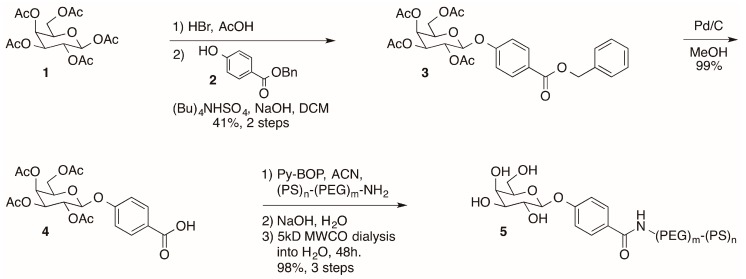
Synthesis of modified di-block co-polymer for nanoparticle assembly. Abbreviations: DCM, dichloromethane; ACN, acetonitrile; MWCO, molecular weight cutoff.

The D-galactose-modified di-block co-polymer required for nanoparticle assembly (**5**) was prepared from β-D-galactose pentaacetate in six steps (Scheme 1). In short, β-D-galactose pentaacetate (**1**) was coupled to benzyl 4-hydroxybenzoate (**2**) to provide ester **3**. Removal of the benzyl protecting group by hydrogenolysis provided carboxylic acid **4**, which was coupled to 6.6 kD amine terminated di-block co-polymer [18]. Removal of the acetate protecting groups on the sugar preceded purification of the sugar-modified polymer **1** by dialysis using a 5 kD molecular weight cutoff (MWCO) membrane. The resulting dialyzed D-galactose-modified polymer (**5**) was concentrated by lypohilization and the resulting white powder was characterized by NMR, IR, and MALDI-MS. Having prepared the requisite galactose-modified di-block copolymer **5**, we proceeded with the assembly of polymeric nanoparticles.

### 2.2. Nanoparticle Preparation

Nanoparticles were prepared by flash nanoprecipitation using established methods [18,19] with varying ratios of the modified D-galactose polymer and unmodified di-block co-polymer to provide 100%-, 50%-, and 25%-surface-modified nanoparticles using the parameters described in Table 1. All nanoparticles were prepared using racemic α-tocopherol (vitamin E) as an inert core stabilizer.

**Figure 1:**
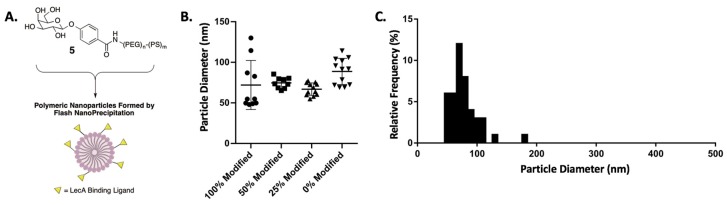
(**A**) Schematic showing assembly of D-galactose surface-modified polymeric nanoparticles. (**B**) Particle size distribution of prepared polymeric nanoparticles. Each data point represents the average particle diameter of an independent formulation. (**C**) Histogram of particle size distribution for all nanoparticles prepared in this study.

All D-galactose nanoparticle (Gal-NP) suspensions were prepared as 0.02 mg/mL solutions in water and characterized in terms of particle diameters (Figure 1) and polydispersity indexes (PDI). The particles prepared are described in this manuscript based on the relative concentration of surface-modified D-galactose to enable direct comparison of the effect of the prepared nanoparticles on lectin binding and biofilm inhibition to free D-galactose, used as a control. Having prepared a series of galactose-modified polymeric nanoparticles, we conducted an evaluation of the lectin binding properties of the prepared particles. 

### 2.3. Inhibition of LecA-Mediated Hemagglutination

The binding affinity of the modified nanoparticles for the *P. aeruginosa* lectin LecA was evaluated using a hemagglutination assay [20]. Briefly, this assay measures the inhibition of LecA-induced hemagglutination of rabbit erythrocytes by comparison with D-galactose as a control. The exceptional effect of ligand multivalency was noted with the 100%- and 50%-modified NP samples, which were evaluated based on the concentration of galactose on the surface of the nanoparticles (Table 2). The strongest effect was observed with the 50%-modified Gal-NP, showing a 992-fold increase in relative potency compared with free galactose. The 100%-modified Gal-NP samples inhibited hemagglutination when modified with concentrations above 6.31μM of galactose, representing a 495-fold increase in potency relative to free galactose. The 25%-modified NP showed no inhibition of hemagglutination up to the highest surface concentration of D-galactose evaluated (2.36 μM). A control 100%-mannose-modified polymeric NP (37.9 μM mannose) showed no inhibition of hemagglutination, supporting the hypothesis that the inhibition of LecA is mediated by specific interactions between the lectin and the nanoparticle surface only with galactose modification.

The high potency of the particles in this assay is consistent with previous reports of high avidity LecA binding using galactose modified dendrimers [12,20]. It is noted that the % modification of the particle leads to maximal inhibition with the 50%-sugar-modified particles. Particles with lower galactose modification (25%-modified) showed no inhibition of hemagglutination at the highest concentrations tested ([galactose] = 2.36 μM) and those with higher modification (100%-modified) had a two-fold higher MIC. It is possible that a sugar concentration greater than 2.36 μM on the nanoparticles is required for high avidity; however, higher sugar concentrations as would be present in the 100%-modified nanoparticles result in the steric inhibition of lectin binding. 

### 2.4. Inhibition of P. aeruginosa PAO1 Biofilm Formation

**Figure 2:**
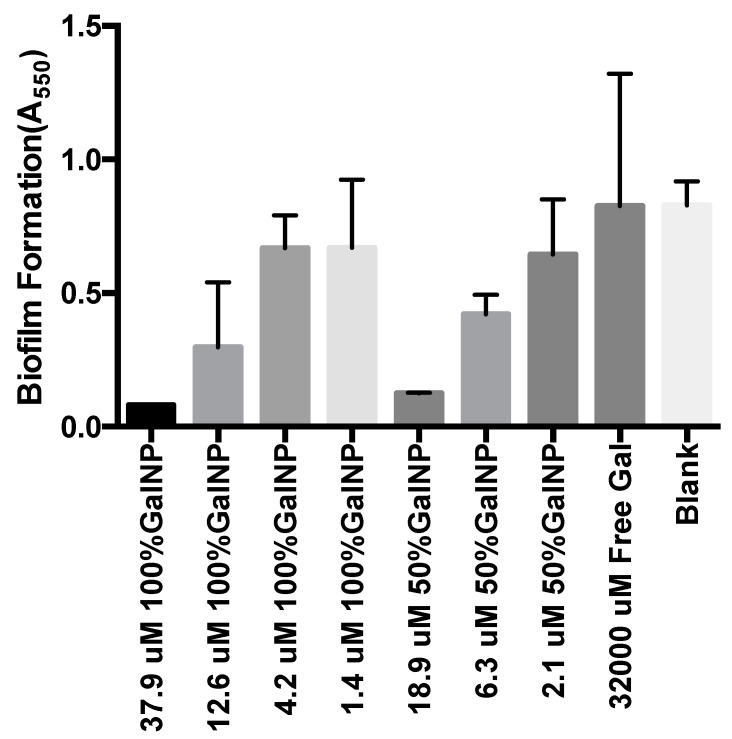
Inhibition of *P. aeruginosa* PAO1 biofilm formation based on the crystal violet assay. Surface-modified Gal-NP samples are described based on the % surface modification and the relative concentration of D-galactose on the nanoparticle surface. All data points are described as the mean of triplicate measurements with error bars representing the standard deviation.

Having identified that our 100%- and 50%-D-galactose-modified polymeric nanoparticles are effective at inhibiting LecA-induced hemagglutination, we evaluated their effect on bacterial biofilm formation using the well-established crystal violet assay [21,22]. Briefly, *P. aeruginosa* strain PAO1 was inoculated in 96-well plates in the presence of varying concentrations of Gal-NP, controls, and/or free D-galactose. Biofilm formation was evaluated after 24 h by removing non-adherent bacteria, crystal violet staining of the adherent cells, and determination of absorbance at 550nm. In this assay, we observed a potent dose-dependent inhibition of biofilm formation with our surface-modified Gal-NP samples (Figure 2). Inhibition of biofilm formation was noted at D-galactose concentrations above 12.6 μM in the 100%-surface-modified nanoparticle samples and at concentrations above 6.3 μM in the 50%-surface-modified series. Again, the significance of ligand valency is evident, as free D-galactose, even at 32,000 μM, showed no inhibition of biofilm formation in this assay.

### 2.5. Evaluation of Growth Inhibition

**Figure 3:**
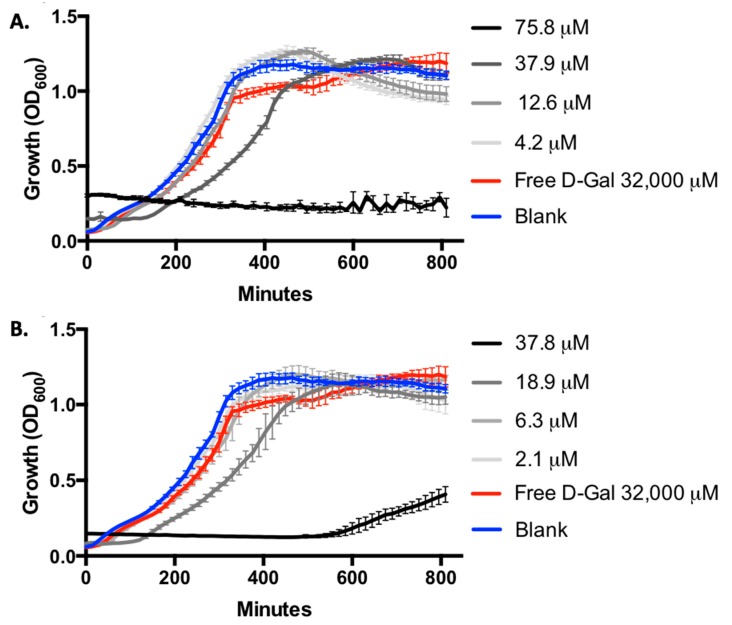
Evaluation of *P. aeruginosa* PAO1 growth by monitoring OD_600_ with 100%-D-galactose-surface-modified nanoparticles (**A**) and 50%-D-galactose-surface-modified nanoparticles (**B**). No significant growth inhibition is observed with 100%-modified Gal-NP at concentrations below 37.9 μM or with 50%-modified Gal-NP at concentrations below 18.9 μM. Growth inhibition was noted at concentrations two-fold higher than those employed in the biofilm inhibition assays (100% Gal-NP at 75.8 μM and 50% Gal-NP at 37.8 μM). Error bars represent standard deviation of triplicate analysis.

To confirm that the potent biofilm inhibitory activity observed was not due to growth inhibition by the nanoparticle samples evaluated, we collected full growth curve data for PAO1 by monitoring OD_600_ (Figure 3). We were surprised to find that the highest concentration of 100%- and 50%-modified nanoparticles (correlating to D-galactose concentrations of 75.8 μM and 37.8 μM, respectively) did potently inhibit bacterial growth. Despite this, no significant inhibition of growth was noted at any of the nanoparticle concentrations evaluated in our biofilm inhibition assay. The absence of growth inhibition supports the hypothesis that that nanoparticle-LecA binding interactions inhibit bacterial biofilm formation via an antivirulence (non-antibiotic) mechanism. 

### 2.6. Evaluation of Biofilm Inhibition and Morphology

We subsequently evaluated the inhibition of biofilm formation in the hyper-virulent isolate of *P. aeruginosa* PA14 by fluorescent confocal microscopy. Briefly, 24-h biofilms were prepared by inoculation of PA14-GFP [23] into Luria broth (LB) containing the appropriate treatments or controls. The resulting biofilm was then evaluated by fluorescent confocal microscopy. In this experiment, we observed clear changes in the overall biomass and average biofilm thickness of the Gal-NP-treated samples, consistent with our observations in the crystal violet assay. Additionally, in this experiment, we captured distinct changes in the morphology of the biofilm that are evident at sub-inhibitory concentrations. These observations further support the inhibition of cellular adhesion by D-galactose surface modified nanoparticles.

**Figure 4:**
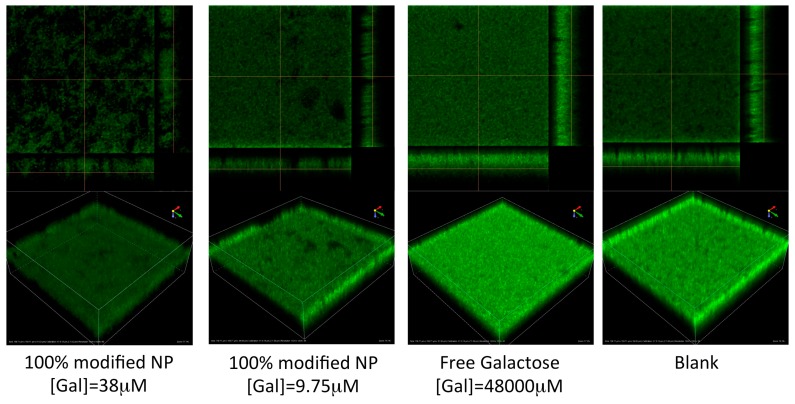
Fluorescent confocal microscopy evaluation of *P. aeruginosa* PA-14 biofilms. Samples treated with Gal-NP show inhibition of biofilm formation. The biofilm morphology at sub-inhibitory concentrations of Gal-NP (e.g., 100%-modified at 9.75 μM) shows evidence of anti-adhesive properties of Gal-NP, which increase with higher concentrations. For additional images see Supporting Information.

**Figure 5:**
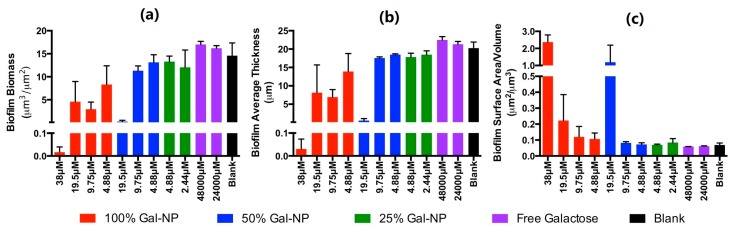
Quantification of (**a**) biofilm total biomass, (**b**) average biofilm thickness, and (**c**) biofilm surface area/volume from fluorescent confocal microscopy analysis. Images were analyzed using Comstat2 processing of duplicate analysis and are reported as means with error bars representing standard deviations.

Within the 100%- and 50%-surface-modified samples of nanoparticles we observed the inhibition of biofilm formation based on evaluation of biofilm biomass and average biofilm thickness at concentrations above ~19.5 μM; however, effects on biofilm morphology were apparent at even lower concentrations (Figure 4). The slightly higher concentrations required to inhibit biofilm formation in the hypervirulent PA14 strain, compared with our crystal violet assay with PAO1, are consistent with previous studies of this bacteria [24,25]. In this assay, we observed no inhibition of biofilm formation by free D-galactose, even at concentrations of 48,000 μM (Figure 4 and Figure 5). Visual inspection of the images collected (Figure 4 and Supporting Information) show that with increasing concentration of D-galactose-modified nanoparticles the biofilm begins to display increasing numbers of spaces within the biofilm structure (Figure 4, panel A vs. B vs. D). This effect is consistent with the anticipated inhibition of cellular adhesion achieved by inhibiting the activity of LecA in the presence of Gal-modified nanoparticles.

Quantification of the biomass and average biofilm thickness of the confocal images using the program Comstat2 shows dose-dependent inhibition of biofilm formation (Figure 5a,b). Most striking is the quantification of biofilm surface area as a function of volume. This calculation nominally provides a measure of the quantity/size of spaces within the biofilm structure. Consistent with the hypothesis that Gal-NP inhibit cellular adhesion without inhibiting growth/viability, we observe a dose-dependent increase in the biofilm surface area/volume (Figure 5c). 

## 3. Discussion

Recently, significant interest has been directed at the development of antivirulence strategies to combat infectious diseases. As these approaches do not directly impact viability or growth, a reduced selective pressure for resistance is anticipated. For the inhibition of biofilm formation, many antivirulence agents being developed focus on the inhibition of the quorum-sensing signaling pathways [14,24,26] or inhibition of bacterial adhesion [20,27,28]. Bacterial lectins are implicated in key roles in bacterial adhesion and biofilm formation, including the lectins LecA and LecB, which play a prominent role in *P. aeruginosa*. Here we report the development of the first LecA-targeted surface-modified polymeric nanoparticle. The particles prepared showed high avidity for LecA in the inhibition of hemagglutination, consistent with a beneficial effect of multivalent display of the lectin ligand on the particle surface. We observed greater than 100-fold enhancement in potency, compared with free D-galactose in the assay with 100%- and 50%-surface modified D-galactose nanoparticles. Further, we demonstrated potent inhibition of *P. aeruginosa* biofilm formation in the presence of these surface-modified nanoparticles, demonstrating the anti-virulence potential of these novel inhibitors of the *P. aeruginosa* lectin LecA.

Previous studies have shown that LecA is cytotoxic [29] and has an important role in cellular adhesion and lung infection [17]. LecA is a homotetramer and displays specificity for galactose. Due to the high affinity of LecA for galactose, numerous multivalent and dendrimer structures have been developed as inhibitors of this protein [12,20,27,28]. The significant increase in LecA binding potency noted in these dendrimeric constructs was captured in the polymeric nanoparticles in this study, which were found to be potent inhibitors of LecA-induced hemagglutination and bacterial biofilm formation. 

Notably, the assembly of nanoparticles as highly multivalent scaffolds for lectin binding avoids many of the synthetic challenges of dendrimer synthesis [30,31]. Accordingly, we anticipate that the application of polymeric nanoparticles to achieve the high lectin-binding potency observed with dendrimers, as demonstrated here, will serve as a powerful and useful complimentary approach for the inhibition of bacterial lectins and other cell-surface receptors. Self-assembly of sugar-modified di-block co-polymers using flash nanoprecipitation [19] further enables ready introduction of multiple different ligands to enable targeting multiple biological targets with a single nanoparticle construct, further enhancing biological activity.

Polymeric nanoparticles have been extensively studied in several medical technologies, especially in the area of drug delivery [11,32,33,34,35,36,37]. The advantages of improved pharmacokinetics and therapeutic properties, including mucus penetration [38], continue to stimulate interest in the field. As demonstrated here, surface modification of the nanoparticle enables targeting of the constructs to the bacterial pathogens of interest, potentially enabling species-specific delivery of drugs, diagnostic reagents, or other small molecules. 

## 4. Conclusions

In conclusion, polymeric nanoparticles synthetically modified to provide surface display of conjugated D-galactose bind to the *P. aeruginosa* lectin LecA with high potency. The benefit of high ligand valency to achieve high avidity lectin binding is efficiently realized in these constructs. Several of the polymeric nanoparticles were found to inhibit biofilm formation in a manner consistent with an antivirulence (anti-adhesion) mechanism of action, providing a new and powerful technology for species-specific inhibition of bacterial virulence. 

## 5. Methods and Materials

### 5.1. General Experimental

Unless otherwise noted, all reactions were performed in flame-dried glassware under an atmosphere of nitrogen or argon using dried reagents and solvents. All chemicals were purchased from commercial vendors and used without further purification. Anhydrous solvents were purchased from commercial vendors.

Flash chromatography was performed using standard grade silica gel 60 230-400 mesh from SORBENT Technologies or using a Biotage Flash Purification system equipped with Biotage SNAP columns. All purifications were performed using gradients of mixtures of ethyl acetate and hexanes. Analytical thin-layer chromatography was carried out using Silica G TLC plates, 200 μm with UV_254_ fluorescent indicator (SORBENT Technologies), and visualization was performed by staining and/or absorbance of UV light. 

NMR spectra for small molecules were recorded using a Varian Mercury Plus spectrometer (400 MHz for ^1^H-NMR; 101 MHz for ^13^C-NMR). Chemical shifts are reported in parts per million (ppm) and were calibrated according to residual protonated solvent. Mass spectroscopy data was collected using an Agilent 1100-Series LC/MSD Trap LC-MS or a Micromass Quattromicro with a Waters 2795 Separations Module LC-MS with acetonitrile containing 0.1% formic acid as the mobile phase in positive ionization mode. Small molecule purity was determined on an Agilent 1100 series equipped with a Phenomenex Kinetex 2.6 µm C18-UPLC column using a gradient of water to acetonitrile with 0.1% TFA.

All final compounds were evaluated to be of greater than 90% purity by analysis of ^1^H-NMR, ^13^C-NMR, and/or analytical HPLC. 

### 5.2. Synthesis of D-Galactose-Modified Polymers

**(2*S*,3*R*,4*S*,5*S*,6*R*)-6-(acetoxymethyl)tetrahydro-2*H*-pyran-2,3,4,5-tetrayl tetraacetate, 2**. D-galactose (5.00 g, 27.75 mmol) was dissolved in acetic acid (185 mL, 0.15 M) at room temperature. Acetyl bromide (16.40 mL, 222.02 mml) was added and the mixture was allowed to stir for 1 h. The resulting solution was washed with toluene (2x, 40 mL), dried over sodium sulfate, and concentrated to dryness. The residue was purified by flash chromatography using a gradient of hexanes to EtOAc to provide (2*S*,3*R*,4*S*,5*S*,6*R*)-6-(acetoxymethyl) tetrahydro-2*H*-pyran-2,3,4,5-tetrayl tetraacetate. Characterization data was consistent with a commercial sample of this compound.

**(2*R*,3*S*,4*S*,5*R*,6*S*)-2-(acetoxymethyl)-6-(4-((benzyloxy)carbonyl)phenoxy)tetrahydro-2*H*-pyran-3,4,5-triyl triacetate, 4.** (2*S*,3*R*,4*S*,5*S*,6*R*)-6-(acetoxymethyl)tetrahydro-2*H*-pyran-2,3,4,5-tetrayl tetraacetate, **2** (2 g, 5.12 mmol) was dissolved in dichloromethane (10 mL, 0.5 M) at room temperature. Hydrobromic acid (33% in AcOH, 1.485 mL) and acetic acid (3.015 mL, 1.14 M) were added and the mixture was allowed to stir. After 30 min, the solution was diluted with 50 mL of DCM, washed with sodium bicarbonate, dried with sodium sulfate, and concentrated to dryness. The residue (2 g) was re-dissolved in DCM (30 mL, 1.5 M) and treated with benzyl-4-hydroxybenzoate, **3** (2.34 g, 10.03 mmol), tetrabutylammonium hydrogensulfate (1.74 mg, 5.12 mmol), and 0.5 M sodium hydroxide (10 mL, 0.5 M). The biphasic mixture was allowed to stir overnight at room temperature, was diluted with DCM and acidified with 1M HCl, extracted with DCM (3 x 50mL), dried over Na_2_SO_4_, and concentrated to dryness. The residue was purified by flash chromatography using a gradient of hexanes to EtOAc to provide (2*R*,3*S*,4*S*,5*R*,6*S*)-2- (acetoxymethyl)-6-(4-((benzyloxy)carbonyl)phenoxy) tetrahydro-2*H*-pyran-3,4,5-triyl triacetate, **4** (1.006 g, 1.848 mmol, 40.76%). ^1^H NMR (400 MHz, CDCl_3_) δ^1^H NMR (400 MHz, CDCl_3_) δ 7.96 (d, *J* = 8.9 Hz, 2H), 7.40–7.22 (m, 5H), 6.95 (d, *J* = 8.4 Hz, 2H), 5.48–5.37 (m, 2H), 5.27 (s, 2H), 5.11–5.01 (m, 2H), 4.20–3.95 (m, 3H), 2.11 (s, 3H), 1.98 (s, 6H), 1.94 (s, 3H). ^13^C NMR (101 MHz, CDCl_3_) δ 170.44, 170.30, 170.19, 169.44, 165.88, 160.46, 136.19, 131.84, 128.72, 128.37, 128.25, 125.14, 116.30, 98.93, 71.39, 70.84, 68.59, 66.93, 66.76, 66.76, 61.50, 20.82, 20.79, 20.76, 20.69, 0.12. ESI-MS: predicted for C_28_H_31_O_12_ [M + H]^+^ 559.544, observed, 559.569.

**4-(((2*S*,3*R*,4*S*,5*S*,6*R*)-3,4,5-triacetoxy-6-(acetoxymethyl)tetrahydro-2*H*-pyran-2-yl)oxy)benzoic acid, 5.** (2*R*,3*S*,4*S*,5*R*,6*S*)-2-(acetoxymethyl)-6-(4-((benzyloxy)carbonyl)phenoxy)tetrahydro-2*H*- pyran-3,4,5-triyl triacetate, **4** (0.719 g, 1.320 mmol) was dissolved in methanol (6.6 mL, 0.2 M) and palladium on carbon (10%, 16 mg, 0.1 mmol) was added to the reaction flask at room temperature. Hydrogen was added via balloon and the reaction was stirred overnight at room temperature. The residue was separated by vacuum filtration washing with methanol to yield 4-(((2*S*,3*R*,4*S*,5*S*,6*R*)-2,3,4,5-tetrahydroxy-6-(hydroxymethyl)tetrahydro-2*H*-pyran-2-yl) methoxy)benzoic acid, **5** (571.2 mg, 1.219 mmol, 61.85%). ^1^H NMR (400 MHz, CDCl_3_) δ 8.05 (d, *J* = 8.3 Hz, 2H), 7.03 (d, *J* = 8.3 Hz, 2H), 5.57–5.43 (m, 2H), 5.20–5.07 (d, *J* = 51.1 Hz, 2H), 4.28–4.06 (d, *J* = 84.7 Hz, 3H), 2.19 (s, 3H), 2.06 (s, 6H), 2.02 (s, 3H). ^13^C NMR (101 MHz, CDCl_3_) δ 171.98, 171.91, 171.38, 171.22, 161.55, 132.97, 132.72, 117.02, 115.98, 99.36, 72.27, 72.16, 70.02, 68.67, 62.60, 49.00, 24.74, 20.69, 20.60, 20.54, 20.50, 20.49, 13.93. ESI-MS: predicted for C_21_H_24_O_12_Na [M + Na]^+^ 491.401, observed, 491.477.

**PS-*b*-PEG-Galactose, 1.** To a room temperature solution of 4-(((2*S*,3*R*,4*S*,5*S*,6*R*)-2,3,4,5-tetrahydroxy-6-(hydroxymethyl)tetrahydro-2*H*-pyran-2-yl) methoxy)benzoic acid, **5** (20.5 mg, 0.068 mmol) in DMF (340uL) was added (Benzotriazol-1-yloxy)tris(dimethylamino)phosphonium hexafluorophosphate (30.1 mg, 0.068 mmol) and the mixture was allowed to stir for 5 min before the addition of PS-*b*-PEG-NH_2_ (150 mg, 0.023 mmol, 6.6 kD, prepared from PS-*b*-PEG-OH as previously described [18]). The resulting solution was stirred at room temperature overnight. The reaction was quenched by the addition of water (1 mL) and EtOH (1 mL), and loaded into a 3.5 kD MWCO dialysis cassette (Pierce^TM^ Slide-A-Lyzer^TM^ G2, Thermo Scientific). The mixture was dialyzed into water for 12 h, at which time the dialysis cassette was transferred to a fresh solution of water and further dialyzed for an additional 12 h. After dialysis, the retained solution was lyopholyzed to provide PS-*b*-PEG-galactose, 1, as a white powder (153.6 mg, quantitative). The product was characterized by MALDI-MS (Bruker MicroFlex LFR MALDI-TOF) in positive linear detection mode using a matrix of dithranol:polymer of 1:1 evaporated from DCM onto a polished steel target. Collected spectra were analyzed with the Flex Analysis software (Bruker); the unmodified PS-*b*-PEG-NH_2_ was evaluated in the same manner as a standard. The observed m/z for PS-*b*-PEG-NH_2_ was 6212.07, and the observed m/z for PS-*b*-PEG-galactose was 6501.40, consistent with complete polymer modification (anticipated Δ m/z for polymer modification = 282.24).

### 5.3. Nanoparticle Assembly

Nanoparticles with defined levels of surface modification were prepared through flash nanoprecipitation (FNP) as previously described [18,19,39]. Briefly, PS, vitamin E (VitE), PS-*b*-PEG, PS-*b*-PEG-galactose, and/or PS-*b*-PEG-mannose were dissolved at defined compositions in THF and rapidly micromixed with an equivalent volume of water within a confined impingement jet mixer, manufactured using HDPE (The Inventors Shop, Cinnaminson, NJ, USA), following the specifications provided [40], and diluted tenfold in a water collection bath. Particles with 0–100%-surface-modification of galactose or mannose were prepared using defined combinations of PS-*b*-PEG/PS-*b*-PEG-galactose or PS-*b*-PEG-mannose. Vitamin E was used as the core bulking material in all NP formulations. Particle sizes were assessed with dynamic light scattering analysis (90Plus Particle Size Analyzer, Brookhaven Instruments Corporation). Size distributions were determined with backscattering measurements from 658-nm illumination and displayed as intensity-weighted distributions; ‘particle diameter’ is the hydrodynamic diameter as calculated by DLS based on the Stokes Einstein equation using instrumental software. The composition of particles formed and particle size characterization data is provided in Table 1.

### 5.4. Hemagglutination Assay

The LecA-induced hemagglutination assay was performed as reported with modifications [20]. LecA (PA-IL) was purchased from Sigma-Aldrich and used as received. Rabbit red blood cells (Innovative Research) at 100% were washed three times with 150 mM NaCl solution then diluted to 5% with NaCl (150 nM) solution. A hemocytometer was used to count the number of red blood cells that were in red blood cell solution and solutions were normalized to 2.94 × 10^14^ cells/mL. To find the minimal full hemagglutination inhibition concentration for LecA, a serial dilution was performed from a stock solution of 1 mg/mL LecA. Briefly, LecA (15 µL) was diluted into Tris buffer (Tris 20 µM, NaCl 100 mM, CaCl_2_ 100 µM, pH = 7.5, 35 µL) in 96-well microtiter V-bottom plates and was subjected to a two-fold serial dilution. Normalized 5% blood solution (10 µL) was added to each well and incubated for 30 min at room temperature. After incubation, the plate was centrifuged for 1 min at low rpm. The minimum full hemagglutination inhibition for LecA was found to be 1.2 µg/mL. Further analysis of inhibition of LecA-induced hemagglutination was performed with LecA at 4xHU (4.8 µg/mL). Briefly, 25 µL of each nanoparticle formulation was subjected to a two-fold serial dilution in Tris buffer and LecA was added to a final concentration of 4xHU. The plate was then incubated for 2 h at room temperature. After incubation, normalized 5% blood solution (10 µL) was added. The plate was then incubated for 30 min at room temperature and then centrifuged for 1 min at low rpm before analysis.

### 5.5. Crystal Violet Biofilm Inhibition Assay

The effect of inhibitors on static biofilm formation was evaluated using the crystal violet staining method with *P. aeruginosa* strain PAO1 [25] following the established procedure [41]. Briefly, overnight cultures of *P. aeruginosa* PAO1 were diluted 1:100 into fresh LB media. Compounds were added at the indicated concentrations in a 96-well microplate before static incubation at 37 °C for 24 h. After incubation, the plates were thoroughly rinsed to remove planktonic cells and the adherent cells were quantified by staining with crystal violet and measurement of A_550_. The assay was performed with five replicates for each compound concentration. After exclusion of the highest and lowest absorbance readings the remaining triplicate readings were described as means with error bars representing standard deviations.

### 5.6. Growth Inhibition Assay

The effect of nanoparticles on bacterial growth was evaluated with *P. aeruginosa* strain PAO1 [25]. Overnight cultures of *P. aeruginosa* PAO1 were diluted 1:100 into fresh LB media. Nanoparticle formulations/compounds were added at the indicated concentrations in a 96-well microplate. The plate was placed in a Molecular Devices SpectraMax i3x incubating plate reader set at 37 °C with shaking. Measurement of A_600_ was recorded every 15 min for the duration of the experiment. Readings are reported as means with error bars representing the standard deviations of triplicate analyses.

### 5.7. Fluorescent Confocal Microscopy

The effect of inhibitors on static biofilm formation was evaluated by confocal fluorescence microscopy with *P. aeruginosa* strain PA14 [25] following the established procedure [42]. Briefly, overnight cultures of PA14-GFP [23] were diluted 1:100 into the designated media. Compounds were added at the indicated concentrations in 96-well microplates with optical grade glass bottoms (Corning) before static incubation at 37 °C for 24 h. After incubation, the samples were imaged on a Nikon Eclipse TI confocal microscope equipped with a 10X lens. Images were acquired from a central point in each well of the microplate. Each compound concentration was evaluated in duplicate. Image stacks were analyzed using Comstat2 [43,44].

## Figures and Tables

**Table 1:** Summary of polymeric nanoparticle (NP) prepared with varying levels of surface modification.

Formulation	Active Stabilizer		Stabilizer		Inert Core		NP Properties	
	Block co-polymer	Conc. (mg/mL)	Block co-polymer	Conc. (mg/mL)	Filler	Conc. (mg/mL)	Z-diameter (nm) ^1^	PDI ^2^
0%	PS-*b*-PEG-Gal	0.00	PS-*b*-PEG	0.40	VitE	0.40	88.83 ± 4.64	0.0327
25% Gal-NP	PS-*b*-PEG-Gal	0.10	PS-*b*-PEG	0.30	VitE	0.40	73.78 ± 2.23	0.0132
50% Gal-NP	PS-*b*-PEG-Gal	0.20	PS-*b*-PEG	0.20	VitE	0.40	74.74 ± 2.26	0.0083
100% Gal-NP	PS-*b*-PEG-Gal	0.40	PS-*b*-PEG	0.00	VitE	0.40	81.47 ± 12.75	0.2693

^1^ Data represent the mean with error described as the standard error of means of at least three independent preparations of the particles, each with three instrumental replicates. ^2^ Polydispersity Index (PDI) = (standard deviation/mean diameter)^^2^. Abbreviations: VitE, vitamin E (α-tocopherol).

**Table 2:** Inhibition of *P. aeruginosa* LecA-induced hemagglutination.

Entry	Formulation	Hemagglutination Assay MIC (uM) ^1^	Relative Potency ^4^
1	D-galactose	3125.0	1.0
2	100%-Modified D-galactose-NP	6.31	495.2
3	50%-Modified D-galactose-NP	3.15	992.1
4	25%-Modified D-galactose-NP	none ^2^	-
5	100%-Modified mannose-NP	none ^3^	-

^1^ MIC = minimal inhibitory concentration for the hemagglutination assay following two-fold serial dilutions of tested compounds; the MIC corresponds to the highest dilution resulting in the inhibition of LecA-induced hemagglutination based on the relative sugar concentration. ^2^ No inhibition of hemagglutination up to highest concentration evaluated (galactose concentration of 2.36 μM). ^3^ No inhibition of hemagglutination up to highest concentration evaluated (maltose concentration of 37.9 μM). ^4^ Relative potency based on concentration of galactose = (MIC_(D-galactose)_/MIC_(ligand)_).
